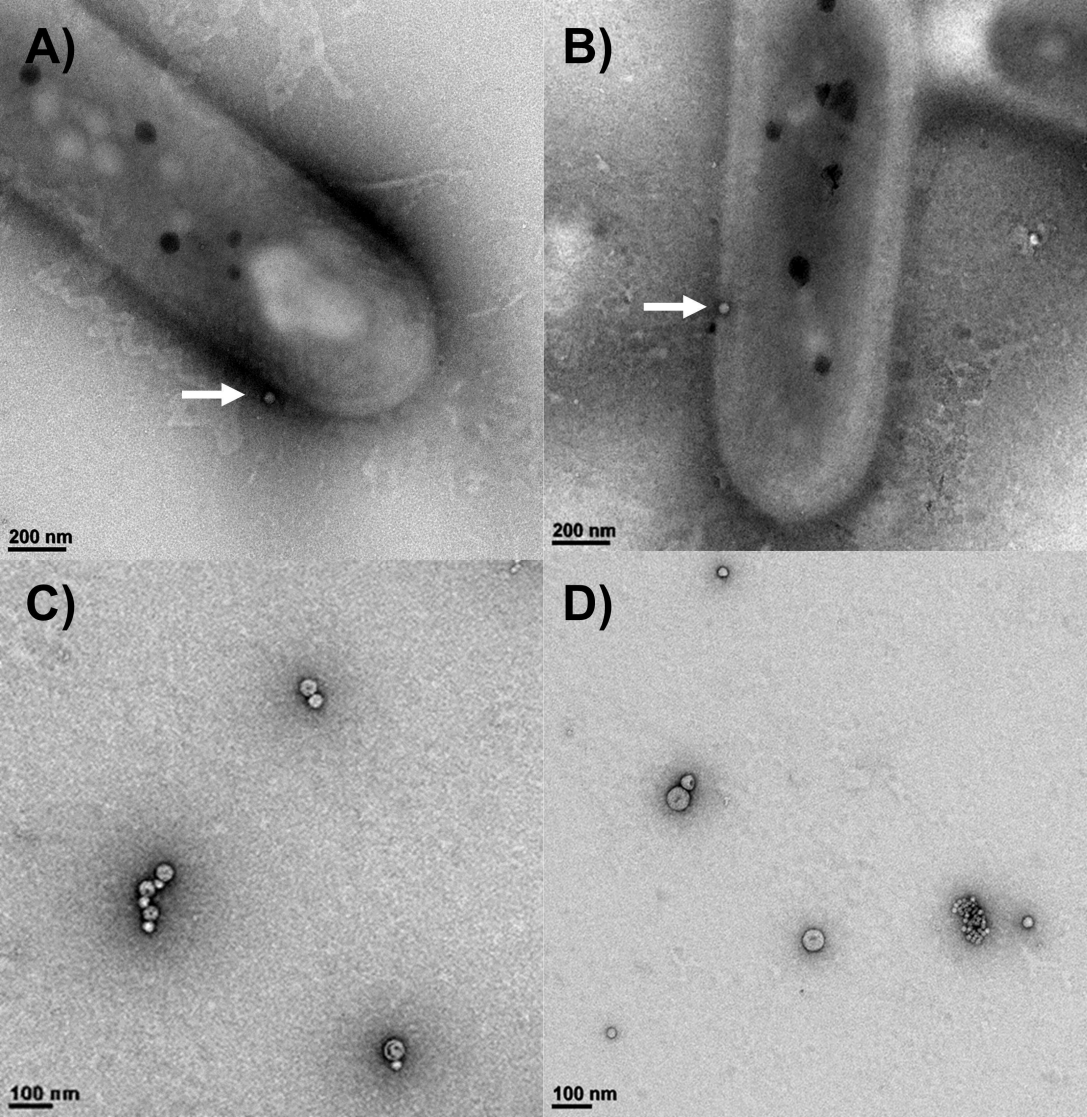# Articles of Significant Interest in This Issue

**DOI:** 10.1128/aem.01395-25

**Published:** 2025-07-23

## AN ENZYMATIC SHORT-CIRCUIT TO THE NITROGEN CYCLE 

Hird et al. (e00292-25) review the microbial enzyme that converts nitrite to ammonium during the dissimilatory reduction of nitrate to ammonium (DNRA) to outcompete denitrification and retain valuable N in agricultural lands.

 
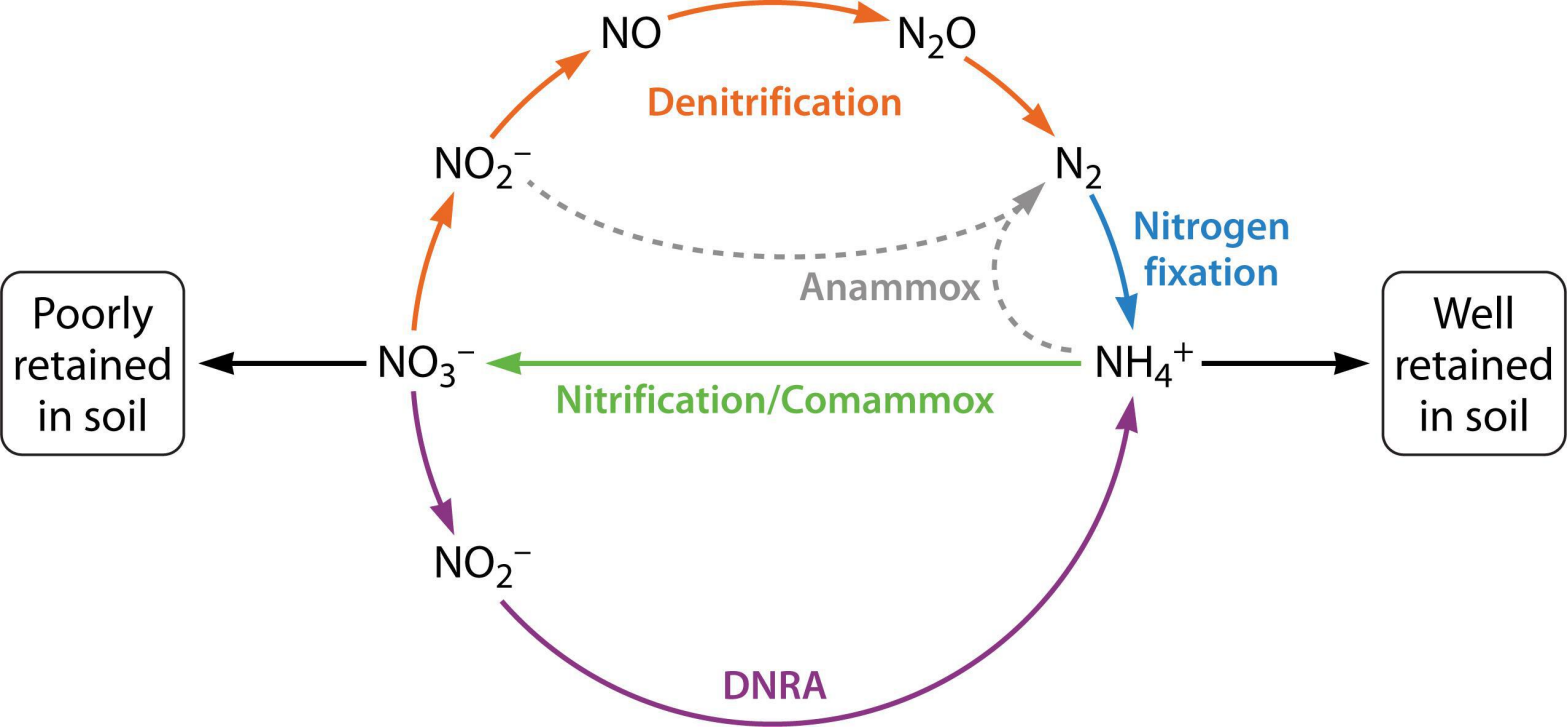


## ANTIBIOTIC-FREE TREATMENT OF BOVINE MASTITIS 

Mastitis is the most important disease impacting the dairy industry and the welfare of dairy cattle. A formulation of bacteriocins and peptidoglycan hydrolases described in this article by Kranjec et al. (e02433-24) provided an effective treatment against bacteria causing bovine mastitis. 


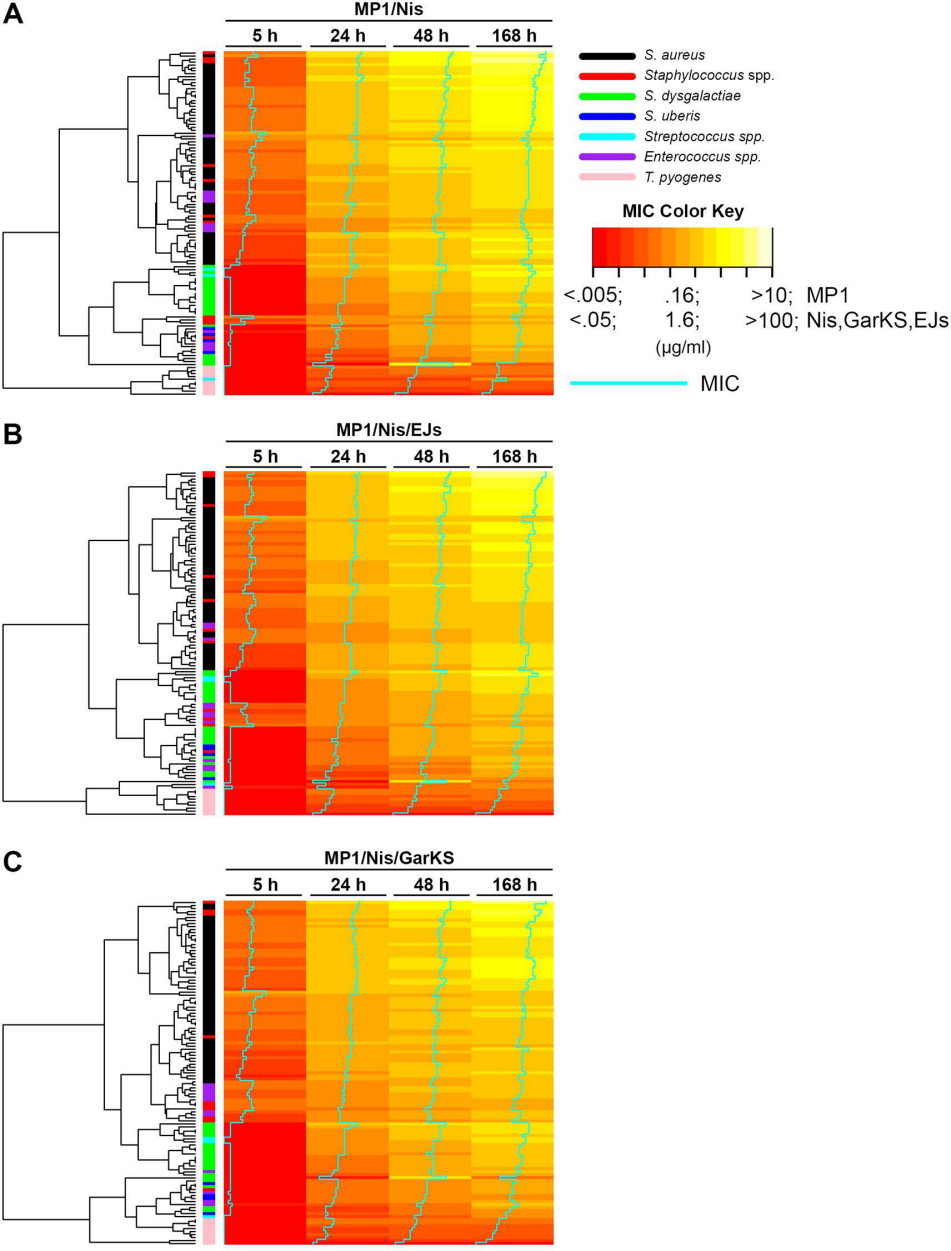


## HUMAN PAPILLOMAVIRUS MEETS WASTEWATER SURVEILLANCE

Wastewater-based surveillance of human papillomavirus (HPV) by Giesbrecht and colleagues (e00348-25) revealed the widespread circulation of high-risk HPV types, including some targeted by vaccines. This is an important approach to inform cancer prevention strategies and evaluate the impact of vaccination programs.


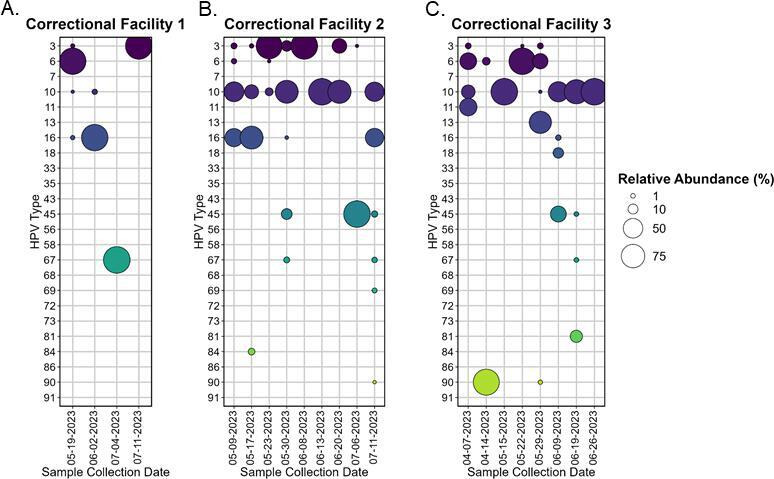


## MICROBIAL THERAPIES FOR WEIGHT LOSS 

Fecal microbiota transplantation (FMT) from healthy donors may provide metabolic benefits to treat obesity. This study by Ruan et al. (e00120-25) illustrates that specific donor-derived taxa are needed for successful engraftment and weight loss. 


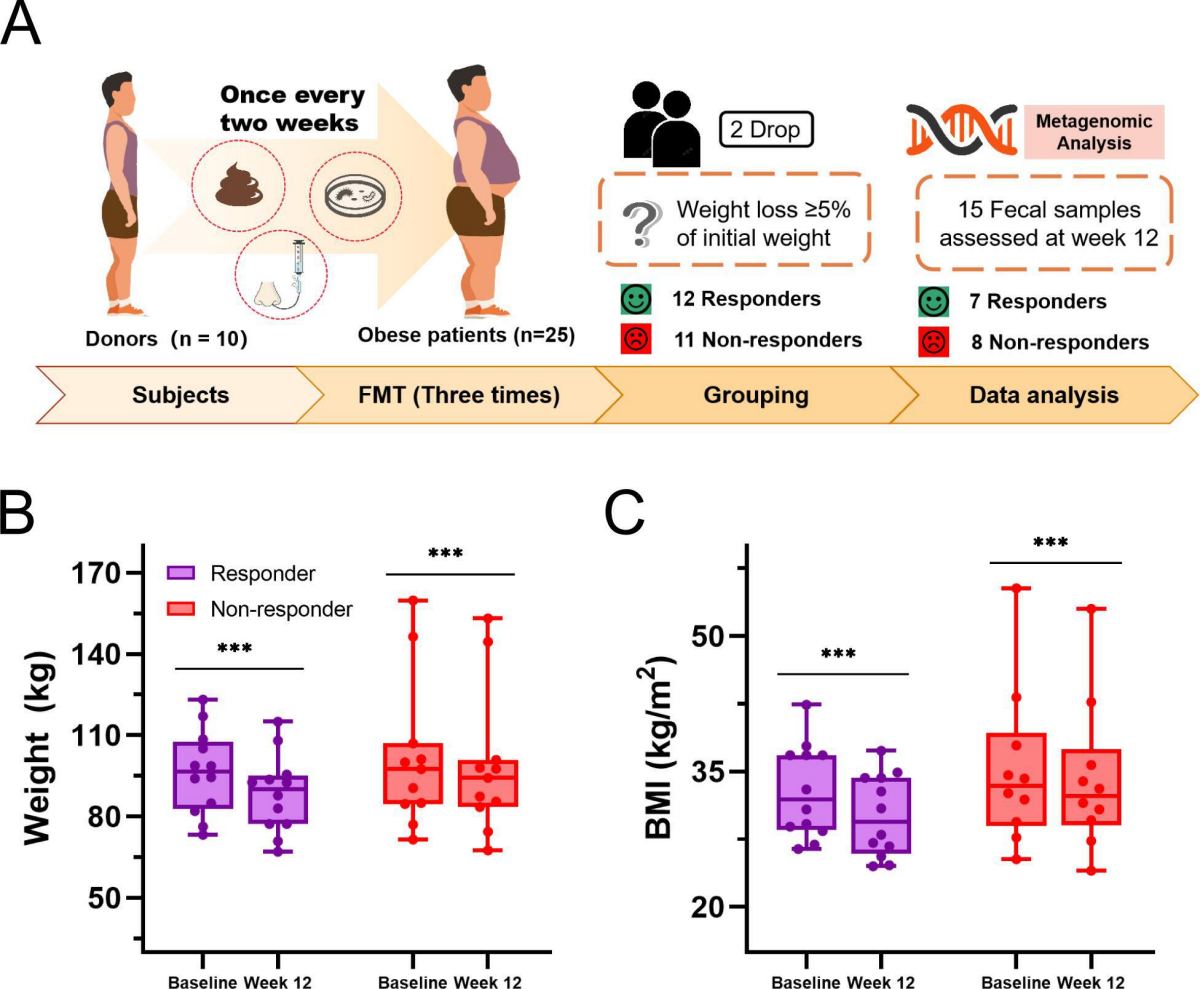


## NO ANTIBIOTIC OINTMENT FOR SICK CORALS

This study by Pearson-Lund et al. (e02407-24) reveals that an amoxicillin ointment applied to lesions in infected corals can disrupt the natural microbiome of adjacent tissues, allowing for opportunistic *Vibrio* species to infect. 


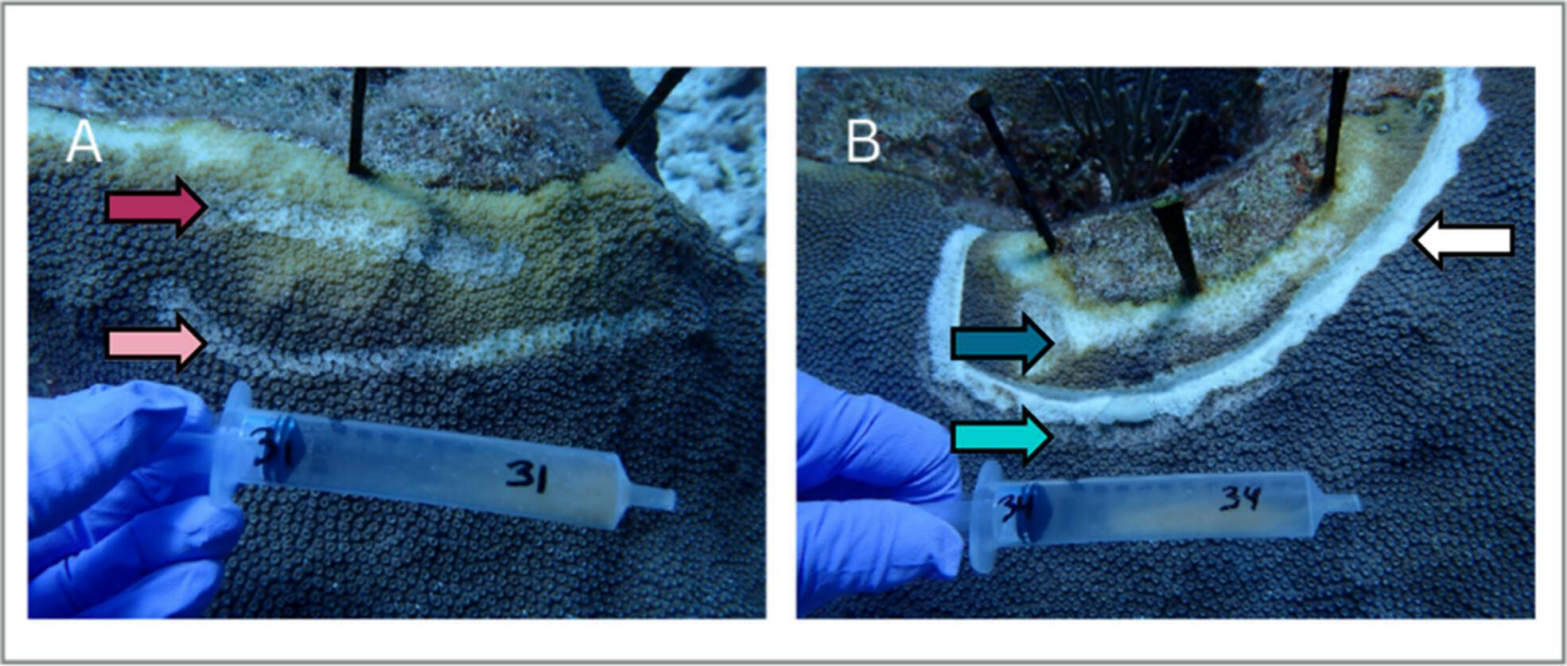


## OF LEAD AND GUTS

Tao et al. (e00372-25) review the impacts of lead exposure on the gastrointestinal tract and discuss dietary interventions for mitigating or preventing the metal’s toxicity. 


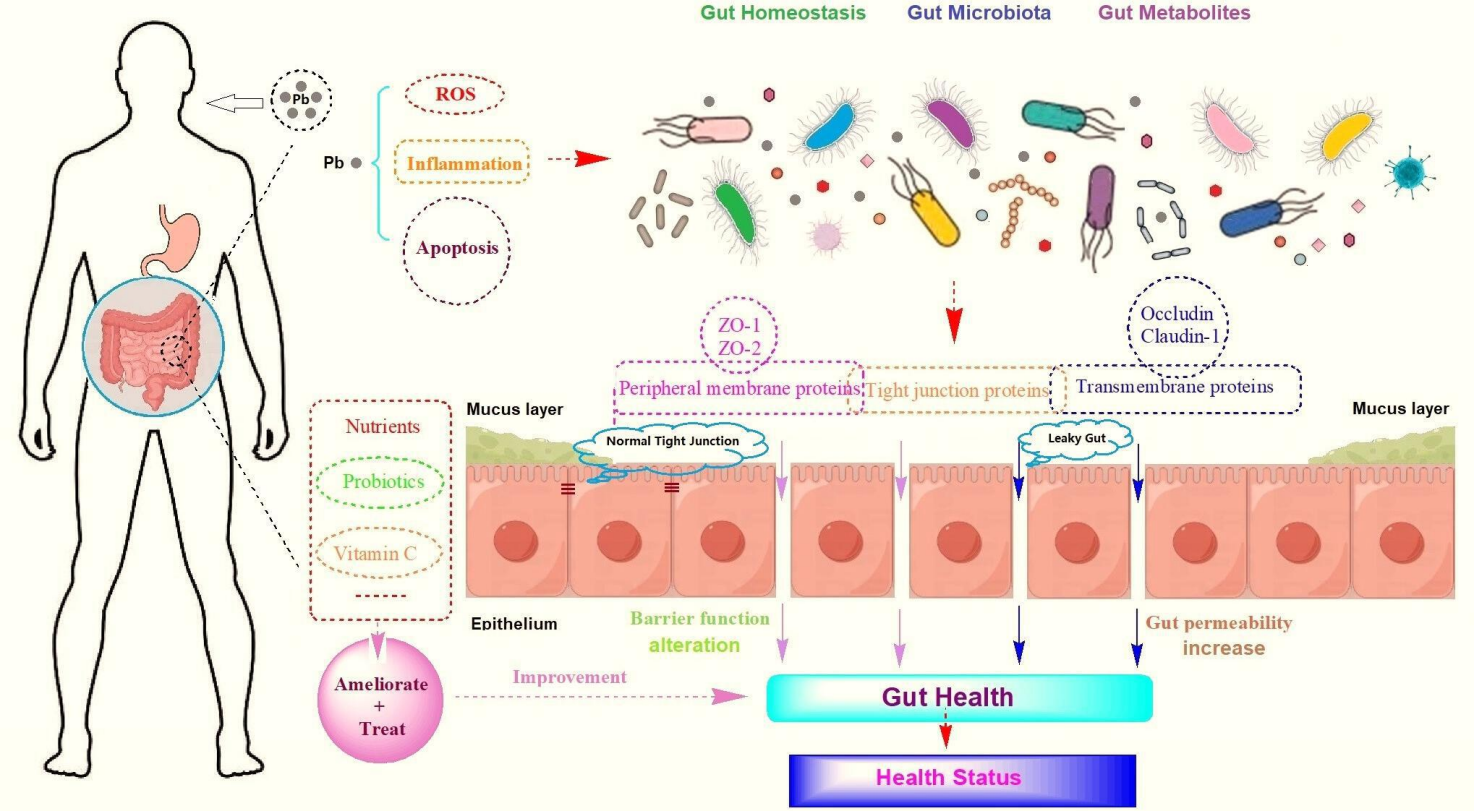


## OUTER MEMBRANE VESICLES WITH A TASTE FOR SULFUR 

Outer membrane vesicles play numerous roles in bacteria. In this study by Noundou et al. (e01019-25), vesicles in anaerobic, thermophilic green sulfur bacteria pack elemental sulfur to exchange the biogenic cargo with other cells.